# Lymphocytic Reaction Combined with MRI Deep Learning Radiomics in Risk Stratification of Distant Metastases in Rectal Cancer

**DOI:** 10.3390/cancers18183020

**Published:** 2026-09-17

**Authors:** Chao Sun, Songli Shi, Jie Sun, Feng Wei, Qian Feng, Liang Yang, Yiming Li

**Affiliations:** 1 Department of Radiology, Tianjin Union Medical Center, The First Affiliated Hospital of Nankai University, Tianjin 300121, China; sunchao@umc.net.cn (C.S.); weifeng@umc.net.cn (F.W.);; 2 Department of Pathology, Tianjin Union Medical Center, The First Affiliated Hospital of Nankai University, Tianjin 300121, China; shisongli@umc.net.cn (S.S.); sunjie@umc.net.cn (J.S.);

**Keywords:** rectal cancer, distant metastases, tumor lymphocytic reaction, radiomics, deep transfer learning

## Abstract

**Objectives:** Our aim was to develop an optimal model for predicting distant metastasis (DM) in rectal cancer (RC) via integrating tumor lymphocytic reaction (LR) with deep learning radiomic (DLR) features. **Methods:** A total of 190 patients with RC were divided into a training cohort (*n* = 133) and a validation cohort (*n* = 57) in a 7:3 ratio. Preoperative radiomics (Rad), deep transfer learning (DTL), and DLR features were extracted from T_2_WI scans, and clinicopathological variables were collected. All models were constructed using the support vector machine (SVM) algorithm, and their performance was evaluated using the area under the receiver operating characteristic curve (AUC). A 3-year follow-up was conducted to analyze 3-year distant-metastasis-free survival (DMFS) outcomes and DM risk. **Results:** Multivariate analysis confirmed LR as an independent predictor of 3-year DMFS (*p* < 0.05). The fusion model integrating LR and DLR exhibited preliminary predictive performance, with AUC values of 0.911 and 0.884 in the training and validation cohorts, respectively. Subgroup observational analyses showed apparent DMFS differences associated with adjuvant chemotherapy exposure among low-risk patients, while such survival patterns were not evident in the high-risk subgroup defined by the nomogram cut-off of 0.3. A moderate negative correlation was detected between DLR signatures and LR (*r* = −0.44, *p* < 0.001), providing preliminary immune-related clues for interpreting deep learning radiomic biomarkers. **Conclusions:** The multimodal fusion nomogram combining pathological LR and DLR signatures shows encouraging preliminary predictive performance for 3-year DMFS risk in patients with RC. This postoperative multimodal tool may provide a preliminary reference for clinicians to implement individualized postoperative surveillance for patients with RC, and the inverse association between DLR and LR helps reveal the immune-related background of imaging signatures.

## Simple Summary

Identifying patients with rectal cancer at high risk of distant spread remains a major clinical challenge. In this retrospective study, we combined pathological immune information called lymphocytic reaction with magnetic resonance imaging-derived deep learning features to build a multimodal prediction tool. Our fusion nomogram exhibited good performance in predicting 3-year distant-metastasis-free survival. Observational survival differences associated with adjuvant chemotherapy exposure were observed within risk subgroups defined by the nomogram. This postoperative multimodal predictive tool may provide a preliminary reference for clinicians to implement individualized surveillance for patients with rectal cancer.

## 1. Introduction

RC is characterized by an insidious onset and the absence of specific early-diagnostic biomarkers, factors that contribute to delayed diagnosis and its rising incidence in China. It has become the second leading cause of cancer-related mortality [1,2]. Novel therapeutic regimens have changed the clinical management of RC, reducing recurrence rates in specific patient subgroups and moderately improving overall prognosis. However, a key clinical challenge persists: even after standard neoadjuvant chemoradiotherapy followed by radical resection, 17–25% of patients develop DM [3], and DM accounts for most disease-related deaths among patients with RC [4,5]. Therefore, accurate evaluation of metachronous DM risk to guide individualized therapy and optimize surveillance plans is an urgent clinical need.

Host immune responses within the tumor microenvironment (TME) regulate tumor initiation and progression [6]. Histological LR, which reflects the host’s antitumor immune response, can be assessed via four indicators: Crohn’s-like LR, peritumoral LR, intratumoral periglandular reaction, and tumor-infiltrating lymphocytes [7]. Existing studies have shown that higher lymphocytic infiltration, abundant tumor-infiltrating lymphocytes, and memory T cells correlate with better survival outcomes in patients with RC [8].

MRI is the standard imaging tool for evaluating local staging and risk factors of RC. Radiomic analyses based on MRI can display intratumoral heterogeneity. Radiomic signatures are associated with tumor invasiveness, treatment response, and patient prognosis [9,10,11]. However, the performance of radiomic approaches for predicting the risk of DM remains limited. Additionally, the inherent complexity, poor stability, and limited interpretability of these radiomic features hinder their successful translation into clinical practice.

In this study, we investigated the association between LR and DM outcomes in RC. Meanwhile, we conducted an in-depth analysis of the correlation between LR and DLR to clarify the biological meaning of DLR features. Based on this correlation, we further systematically compared the predictive performance of five groups for DM risk and for identifying chemotherapy-associated survival subgroups: TME-related LR biomarkers, MRI Rad, DTL, DLR, and the fusion model. In conclusion, this study aimed to establish a prediction model combining pathological LR and imaging-derived DLR signatures for DM risk, with balanced clinical practicality and predictive performance.

## 2. Methods

### 2.1. Patients

**Figure 1:**
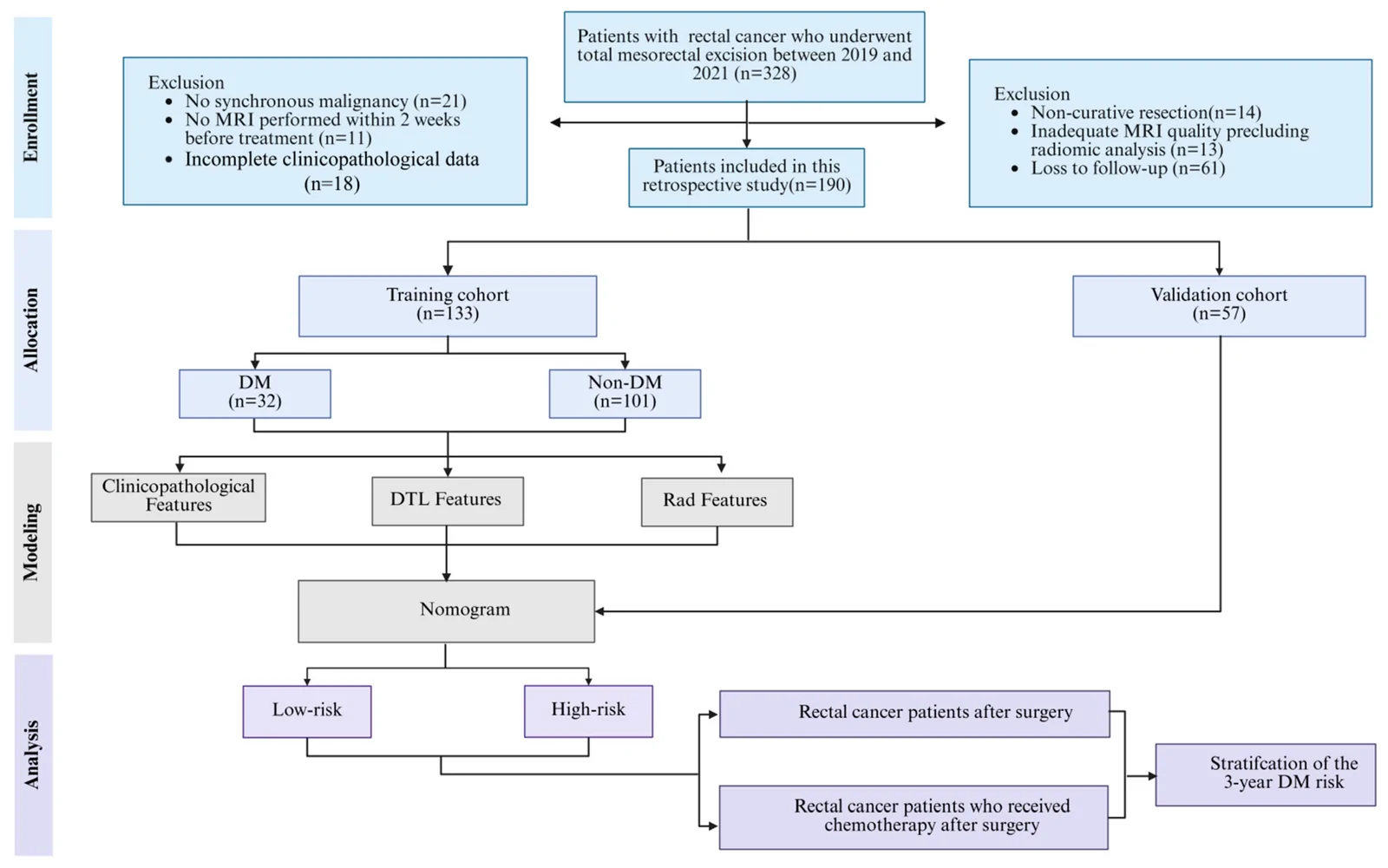
Flowchart of patient inclusion and exclusion in this study.

A retrospective analysis was performed on data from 328 consecutive patients with RC treated at Tianjin Union Medical Center from January 2019 to December 2021. After screening according to this study’s inclusion and exclusion criteria, 190 patients were ultimately enrolled, and the detailed participant selection workflow is shown in Figure 1. Of note, this validation cohort was randomly generated from the same single-center patient pool, corresponding to internal hold-out validation rather than independent external validation from separate institutions or different time periods.

Clinicopathological data were collected for all patients, including age, sex, serum carcinoembryonic antigen (CEA) levels, and postoperative chemotherapy. All participants underwent postoperative follow-up every 3 months during the first year, with semi-annual visits scheduled subsequently. The minimum total follow-up duration was 3 years. DM was defined as extrapelvic tumor recurrence confirmed by imaging or histopathological examination. The primary survival endpoint was DMFS, defined as the time from radical surgery to the first occurrence of distant metastasis. Deaths occurring without preceding distant metastasis were treated as censored observations. Patients who did not experience an endpoint event were censored at their last follow-up.

Postoperative adjuvant chemotherapy regimens included three fluoropyrimidine-based schedules: monotherapy with 5-fluorouracil (5-FU), combination of 5-FU and leucovorin (5-FU/LV), and capecitabine plus oxaliplatin (CapeOx). No standardized prospective protocol for adjuvant chemotherapy was available; treatment selection was at the discretion of the treating clinicians.

### 2.2. Workflow

**Figure 2:**
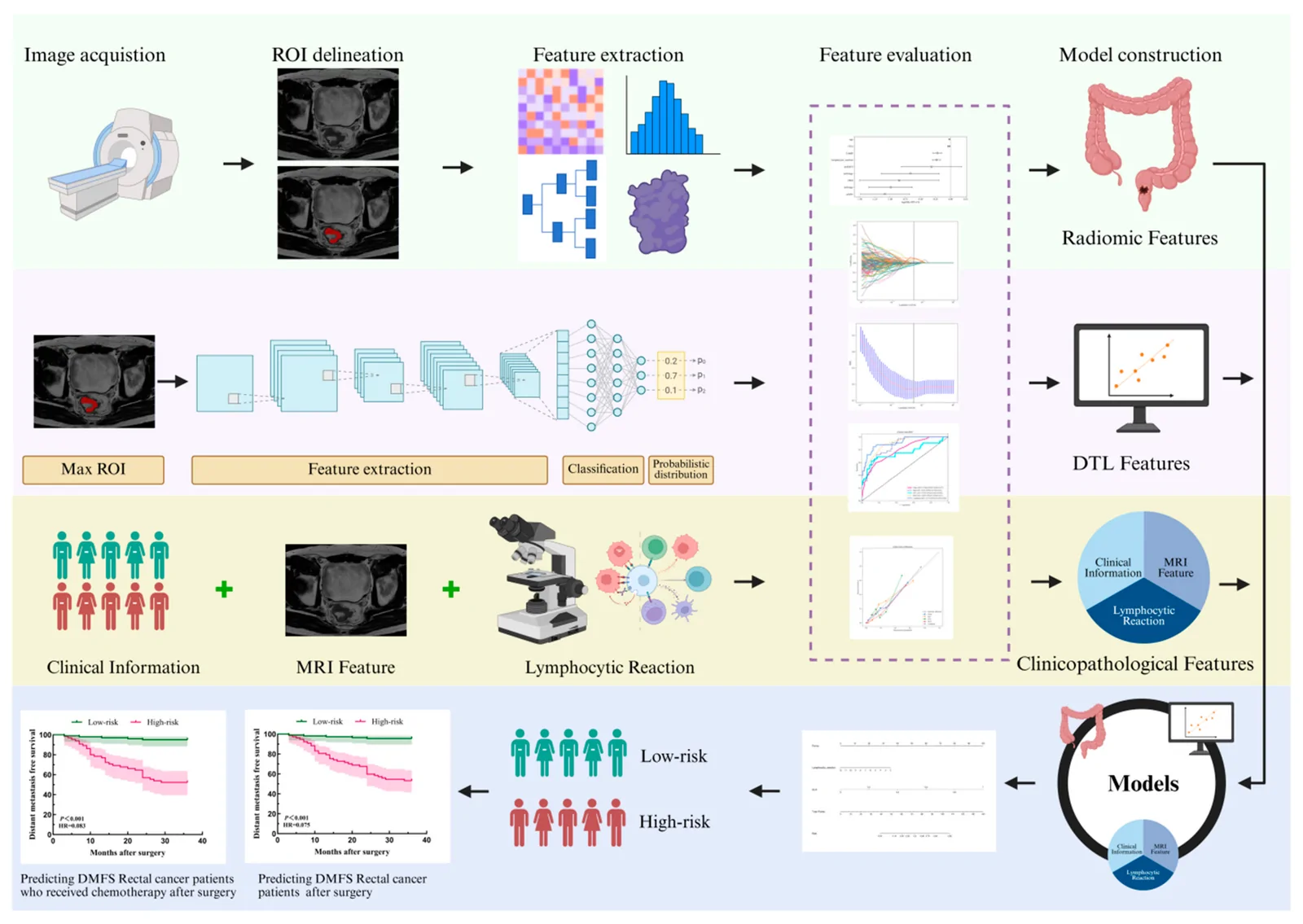
Schematic representation of this study’s design and procedural steps. The workflow includes pretreatment-MRI image acquisition, region-of-interest delineation, hand-crafted radiomic feature extraction, and deep-learning-based DTL feature extraction. Clinical information, MRI-derived features, and histopathological lymphocytic-reaction status were integrated to generate clinicopathological features. After feature evaluation and screening, five independent predictive models were built: a clinicopathological model, radiomic feature model, DTL deep learning feature model, DLR feature model, and final fusion combined model. The fusion model was applied to stratify patients with RC into low-risk and high-risk subgroups. Kaplan–Meier survival analysis was further performed to evaluate DMFS among patients with and without postoperative chemotherapy. (Created with BioRender.com.)

Figure 2 illustrates the workflow in this study.

### 2.3. Histopathological Evaluations

Formalin-fixed paraffin-embedded tumor tissue blocks were collected from patients with RC receiving surgical resection at Tianjin Union Medical Center. Two pathologists blinded to all clinical and imaging data reviewed hematoxylin–eosin-stained sections following published protocols and recorded histopathological results, including four LR subcomponents [7]. Discrepancies between the two observers were resolved through joint discussion to reach a consensus.

**Figure 3:**
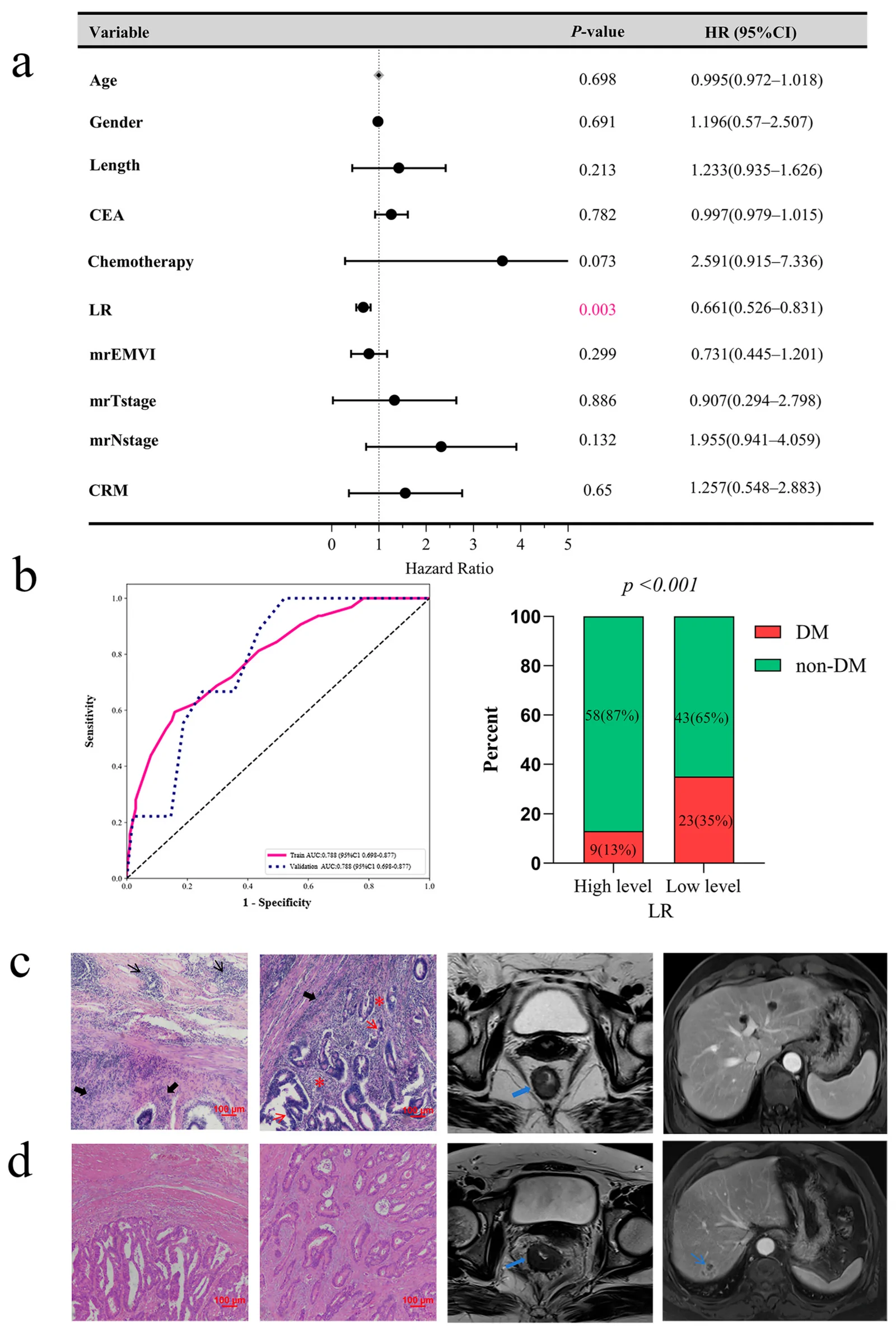
Multivariate Cox regression analysis, predictive performance of LR, and representative multimodal images. (**a**) Forest plot of multivariate Cox regression analysis for DMFS, presenting hazard ratios and 95% confidence intervals for each clinical–radiological variable. (**b**) (**Left**) ROC curves illustrating the predictive performance of LR for distant metastasis risk in the training (solid pink line) and validation (dotted blue line) cohorts. (**Right**) Stacked bar chart comparing the proportions of patients with DM (red) and non-DM (green) between high-level and low-level LR subgroups. (**c**,**d**) Representative hematoxylin–eosin (H&E)-stained histopathological sections and pretreatment MRI images. (**c**) A high-LR patient without distant metastasis occurrence. (**d**) A low-LR patient who developed DM. Black thin arrows indicate Crohn’s-like LR; thick black arrows indicate peritumoral LR; red arrows denote tumor-infiltrating lymphocytes; asterisks mark intratumoral periglandular reaction. Thick blue arrows indicate the rectal primary tumor, and thin blue arrows denote liver metastatic lesions.

Four LR subcomponents were evaluated: Crohn’s-like LR, peritumoral LR, intratumoral periglandular reaction, and tumor-infiltrating lymphocytes. The four markers are marked in Figure 3c,d as follows: (1) Crohn’s-like LR (black arrows); (2) peritumoral LR (thick black arrows); (3) tumor-infiltrating lymphocytes (red arrows); (4) intratumoral periglandular reaction (asterisks).

Crohn’s-like LR refers to transmural lymphoid aggregates; peritumoral LR denotes discrete lymphoid clusters surrounding the tumor; intratumoral periglandular reaction represents lymphocytic infiltration within intratumoral stroma; tumor-infiltrating lymphocytes indicate lymphocytes residing in tumor epithelial regions. Based on published scoring standards, each of the four LR subcomponents was scored separately on a four-tier scale: 0, 1+, 2+, and 3+. A weighted composite LR score was computed from the four subcomponent scores. Component weights were obtained from multivariate Cox proportional hazards regression fitted exclusively on the training cohort, with 3-year DMFS as the survival endpoint. Hazard ratios estimated from this training set Cox model were used as weights for each LR subcomponent. No samples from the validation cohort entered weight estimation to prevent data leakage.

Within the training cohort, receiver operating characteristic (ROC) analysis for 3-year DMFS was performed on the resulting weighted composite LR score. The optimal dichotomization threshold was identified by maximizing the Youden index, using only training cohort data. The training-set-derived optimal cutoff value was 5. Patients with weighted composite LR score ≥ 5 were defined as high-LR, whereas patients with weighted composite LR score < 5 were defined as low-LR.

### 2.4. Acquisition and Preprocessing of MRI Data

MRI examinations were performed using either a 1.5 T scanner (Achieva, Philips Healthcare, Best, The Netherlands) or a 3.0 T scanner (Skyra, Siemens Healthcare, Erlangen, Germany). The high-resolution axial T_2_WI sequence used in this study was Turbo Spin Echo without fat saturation. Detailed information is presented in Supplementary Table S1. Images were resampled to an isotropic voxel size of 1 × 1 × 1 mm^3^ using the nearest neighbor interpolation method.

### 2.5. MR Image Evaluations

Two senior radiologists with 12 and 20 years of MRI experience completed tumor imaging evaluation on a Picture Archiving and Communication System (PACS) workstation. MRI-derived evaluation indicators covered total tumor length, T stage, N stage, extramural vascular invasion (EMVI), and circumferential resection margin (CRM). All inconsistent interpretations were unified via joint consensus discussion.

### 2.6. Tumor Segmentation

One radiologist with 12 years of rectal MRI experience performed semi-automatic segmentation using open-source ITK-SNAP software (version 3.8.0) (ww w.itk-snap.org (accessed on 20 June 2024)).Segmentation covered the full rectal wall lesion on high-resolution axial T_2_WI, while ambiguous normal rectal tissue and mucosal edema were excluded. To ensure stable radiomic feature extraction, all segmentation masks were reviewed by a senior radiologist, and disagreements were settled via joint discussion.

Given that the DTL model required rectangular images enclosing the complete tumor region of interest (ROI), we selected the largest axial tumor slice from each patient as model input.

### 2.7. Feature Extraction

We extracted 1198 conventional radiomic features from the 3D ROI via PyRadiomics (version 2.1.0), with full details listed in Supplementary File S1. To mitigate overfitting induced by limited training samples, we adopted a transfer learning framework. A prebuilt ResNet50 convolutional neural network was then applied to extract 2048 DTL features, and relevant feature details are documented in Supplementary File S2. For the DTL pipeline, ResNet50 weights pretrained on ImageNet-1k from the PyTorch o (version 1.13.1, https://pytorch.org (accessed on 20 June 2024))fficial repository were utilized. The entire classification head was discarded, and convolutional layers were frozen to serve as a fixed feature extractor without fine-tuning. For each patient, the single largest axial tumor slice was cropped with a 10-pixel margin surrounding the tumor ROI and resized to 224 × 224 pixels. Single-channel grayscale MRI slices were replicated across three channels to generate three-channel input tensors compatible with the ImageNet-pretrained ResNet50. No intensity clipping was performed on MRI pixel values, and no data augmentation was applied during feature extraction. The 2048-dimensional DTL features were obtained from the global average pooling layer preceding the final fully connected layer.

### 2.8. Model Construction

Intraclass correlation coefficients (ICCs) were calculated to assess radiomic feature reproducibility; only features with ICCs ≥ 0.75 were kept for downstream analysis. Comprehensive interobserver agreement analyses covering MRI segmentation ICCs, pathological-scoring-weighted kappa coefficients, and discrepancy statistics are presented in Supplementary Table S2. To ensure a fair head-to-head comparison among all predictive models, we adopted a unified modeling pipeline for all feature sets.

The pretrained ResNet50 network was employed solely as a fixed feature extractor, with its intrinsic classification head fully discarded. Feature screening was conducted in three consecutive steps. First, Z-score normalization was performed: per-feature mean and standard deviation were estimated solely on the training cohort. These training-derived normalization parameters were subsequently applied to scale features for both the training cohort and the held-out validation cohort, avoiding any statistical information leakage from validation samples. Second, Spearman’s rank-order correlation analysis was used to eliminate redundant features: for feature pairs with correlation coefficients above 0.9, only one feature was preserved. Third, penalized binomial logistic least absolute shrinkage and selection operator (LASSO) regression (L1 penalty) was performed on the binary endpoint of 3-year distant metastasis status (DM: occurred versus not occurred) to identify predictive imaging features; features with non-zero regression coefficients were retained. The optimal λ hyperparameter was determined by minimizing binomial deviance via 5-fold cross-validation, and all LASSO fitting and λ-optimization steps were strictly confined to the training cohort. All feature-filtering rules including Spearman-correlation-based redundancy removal and the optimal λ for LASSO were determined exclusively within the training cohort; the resulting feature subset was directly applied to validation cohort samples without re-running feature selection.

After screening, support vector machine (SVM) models with radial basis function kernel were constructed for each feature set. Hyperparameters including penalty coefficient C and kernel width γ were optimized via grid search combined with 5-fold cross-validation strictly restricted to the training cohort only, without utilizing any validation-cohort data to avoid data leakage. Model discriminative ability was assessed using ROC curves and AUC values. Bootstrap resampling with 1000 replicates was performed on the training dataset to obtain optimism-corrected AUC estimates; bootstrap-derived 95% confidence interval was calculated for the validation set AUC. The present study utilized internal hold-out validation: all feature filtering, hyperparameter tuning, LR weight fitting and threshold determination procedures were completed exclusively within the training cohort, while the validation cohort was reserved purely for final performance evaluation and was never accessed during any model-building procedure.

### 2.9. Statistical Analysis

Categorical variables were compared using the chi-square test or Fisher’s exact test, while continuous variables were analyzed with the *t*-test or Mann–Whitney U test for baseline patient characteristics. A *p* value < 0.05 was considered statistically significant. IBM SPSS Statistics (version 22.0) was used for clinical variable analyses. ICC calculation, Z-score normalization, and LASSO regression were implemented in Python (version 3.10).

## 3. Results

### 3.1. Patient Characteristics

In total, 190 patients with pathologically confirmed RC were enrolled. Participants were split into a training cohort (*n* = 133) and a validation cohort (*n* = 57) in a 7:3 ratio via a random number table. Within each cohort, patients were stratified into DM and non-DM groups based on the occurrence of DM within the 3-year follow-up period to explore associations between baseline features and DM risk. The exact number of DM events was 32 in the training cohort (*n* = 133) and 9 in the validation cohort (*n* = 57).

The baseline clinicopathological characteristics of the two cohorts are summarized in Table 1. In the training cohort, significant differences in tumor length, tumor LR, mrEMVI, mrN stage, and chemotherapy were identified between the DM and non-DM groups (all *p* < 0.05). In the validation cohort, only tumor LR and mrEMVI differed significantly between the two groups (both *p* < 0.05). Compared with patients without DM, those who developed DM presented distinct clinicopathological features, including longer tumor length, lower tumor LR intensity, higher mrEMVI positivity, advanced mrN stage, and a higher proportion of patients who did not receive postoperative adjuvant chemotherapy. No significant differences in any baseline clinicopathological indicators were found between the training and validation cohorts (all *p* > 0.05), suggesting favorable baseline balance and reliable subsequent analytical results.

### 3.2. Interobserver Agreement of Imaging and Pathological Evaluations

Interobserver reproducibility analysis was performed for MRI tumor segmentation and pathological LR scoring (Supplementary Table S2). For extracted Rad and DTL features, ICC values of Rad features ranged from 0.79 to 0.91 (median = 0.85; mean ± SD = 0.86 ± 0.06), while ICC values of DTL features ranged from 0.78 to 0.90 (median = 0.82; mean ± SD = 0.82 ± 0.04). All candidate features achieved an ICC ≥ 0.75, satisfying the pre-defined reproducibility threshold.

The weighted Kappa coefficients for the four pathological scoring items indicated good interobserver agreement: 0.81 (95% CI: 0.73–0.87) for Crohn’s-like LR, 0.79 (95% CI: 0.69–0.86) for peritumoral LR, 0.84 (95% CI: 0.76–0.90) for intratumoral periglandular reaction, and 0.86 (95% CI: 0.78–0.91) for tumor-infiltrating lymphocytes.

### 3.3. Feature Selection

Multivariate Cox regression analysis demonstrated that LR was the only independent factor influencing DMFS (Figure 3a). Higher LR levels correlated with a lower risk of DM, while lower LR levels were associated with an increased DM risk (Figure 3b–d).

**Figure 4:**
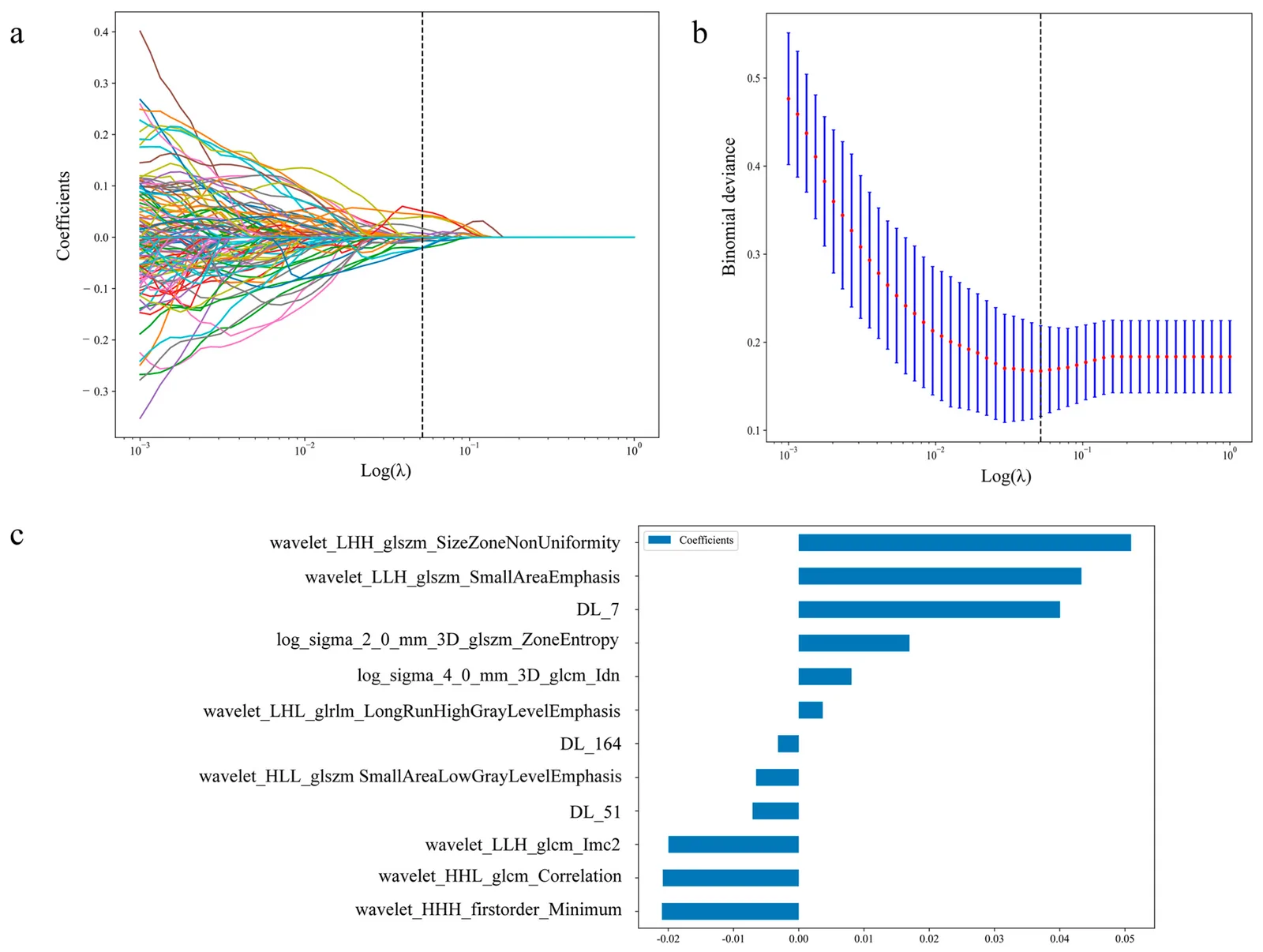
Feature selection using LASSO regression model. (**a**) LASSO coefficient profiles of all candidate imaging features; each colored line represents the coefficient trajectory of one individual feature. Vertical dashed line indicates optimal λ value (λ = 0.0518). (**b**) Binomial deviance curve for selecting optimal λ via cross-validation. Red dots denote binomial deviance for different λ values; vertical blue bars represent standard error. Vertical dashed line corresponds to optimal λ (λ = 0.0518). (**c**) Final set of selected radiomic and deep learning features and their corresponding regression coefficients (blue bars).

The DLR model was constructed by integrating conventional radiomic features and DTL features. After sequential feature screening and optimization, a total of 12 tumor features were retained. These features consisted of nine Rad features and three DTL features. Full names, categories, and LASSO coefficient weights of all 12 selected features are listed in Supplementary Table S3, corresponding to the LASSO coefficient histogram shown in Figure 4. The fusion model was subsequently developed by incorporating the LR indicators into the DLR model framework.

### 3.4. Model Evaluation

**Figure 5:**
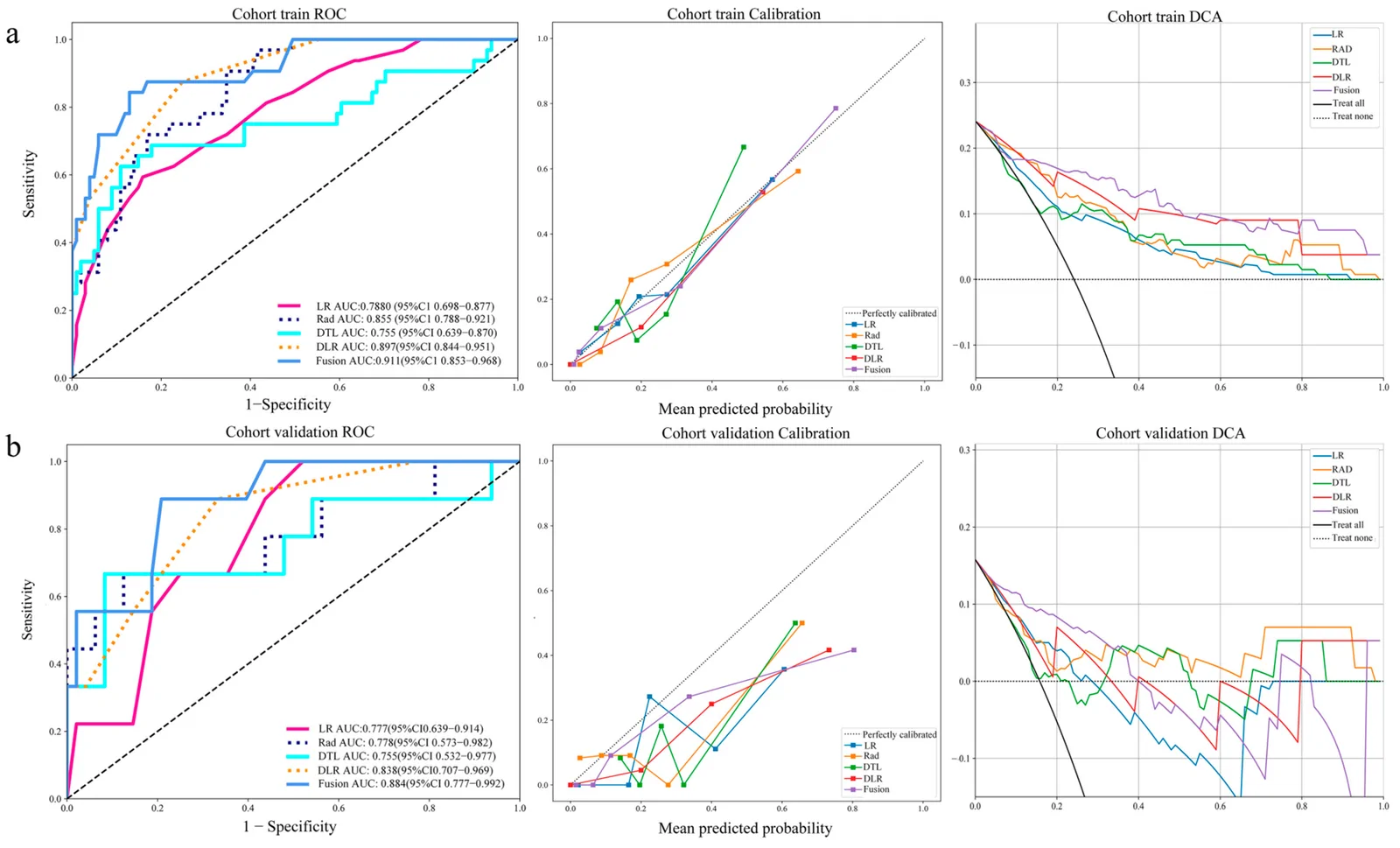
Diagnostic performance of individual signatures and fusion nomogram in training and validation cohorts. (**a**,**b**) ROC curves, calibration curves, and decision curve analysis for LR (pink), Rad (dark blue dotted), DTL (cyan), DLR (orange dotted), and fusion model (blue solid line) in the training (**a**) and validation (**b**) cohorts. From left to right within each panel: ROC curves, calibration curves, DCA curves. From left to right: ROC curves, calibration curves, DCA curves.

The predictive performance of each model was assessed in the training and validation cohorts. The LR model yielded AUC values of 0.788 (95% CI: 0.698–0.877) and 0.777 (95% CI: 0.639–0.914) in the training and validation cohorts, respectively. The DTL model showed comparable predictive efficacy to the LR model, with an AUC of 0.755 in both cohorts (training cohort: 95% CI: 0.639–0.870; validation cohort: 95% CI: 0.532–0.977). The conventional radiomic model achieved better predictive performance than the LR and DTL models, with an AUC of 0.855 (95% CI: 0.788–0.921) in the training cohort and 0.778 (95% CI: 0.573–0.982) in the validation cohort. The DLR model showed further improved predictive performance, outperforming the conventional radiomic, DTL, and LR models, with AUCs of 0.897 (95% CI: 0.844–0.951) and 0.838 (95% CI: 0.707–0.969) in the two cohorts. The fusion model achieved the best predictive performance among all constructed models, with the highest AUC values of 0.911 (95% CI: 0.853–0.968) in the training cohort and 0.884 (95% CI: 0.777–0.992) in the validation cohort. Detailed AUC results and statistical information for all models are shown in Figure 5. Sensitivity, specificity, positive predictive value (PPV), negative predictive value (NPV), and PR-AUC results for all models are summarized in Supplementary Table S4. Bootstrap resampling (1000 replicates) was applied for optimism correction. The optimism-corrected AUC of the fusion model in the training cohort was 0.872 (apparent AUC = 0.911, optimism = 0.039). The 95% confidence interval for the validation set AUC (0.884) was 0.741–0.976.

The calibration curves of the fusion nomogram were plotted to validate the model’s predictive accuracy (Figure 5). The predicted DM risk was generally consistent with the actual observed incidence. Nevertheless, the model exhibited some predictive bias: it slightly underestimated the actual DM risk at threshold probabilities of 30–75%, while overestimating the risk when the threshold probability exceeded 75%.

Decision curve analysis was performed to evaluate the clinical utility of the fusion model (Figure 5). The fusion model yielded a higher net clinical benefit than the two extreme clinical strategies (universal treatment and no treatment), as well as the LR, DTL, and DLR models, supporting its potential value in clinical decision-making.

**Figure 6:**
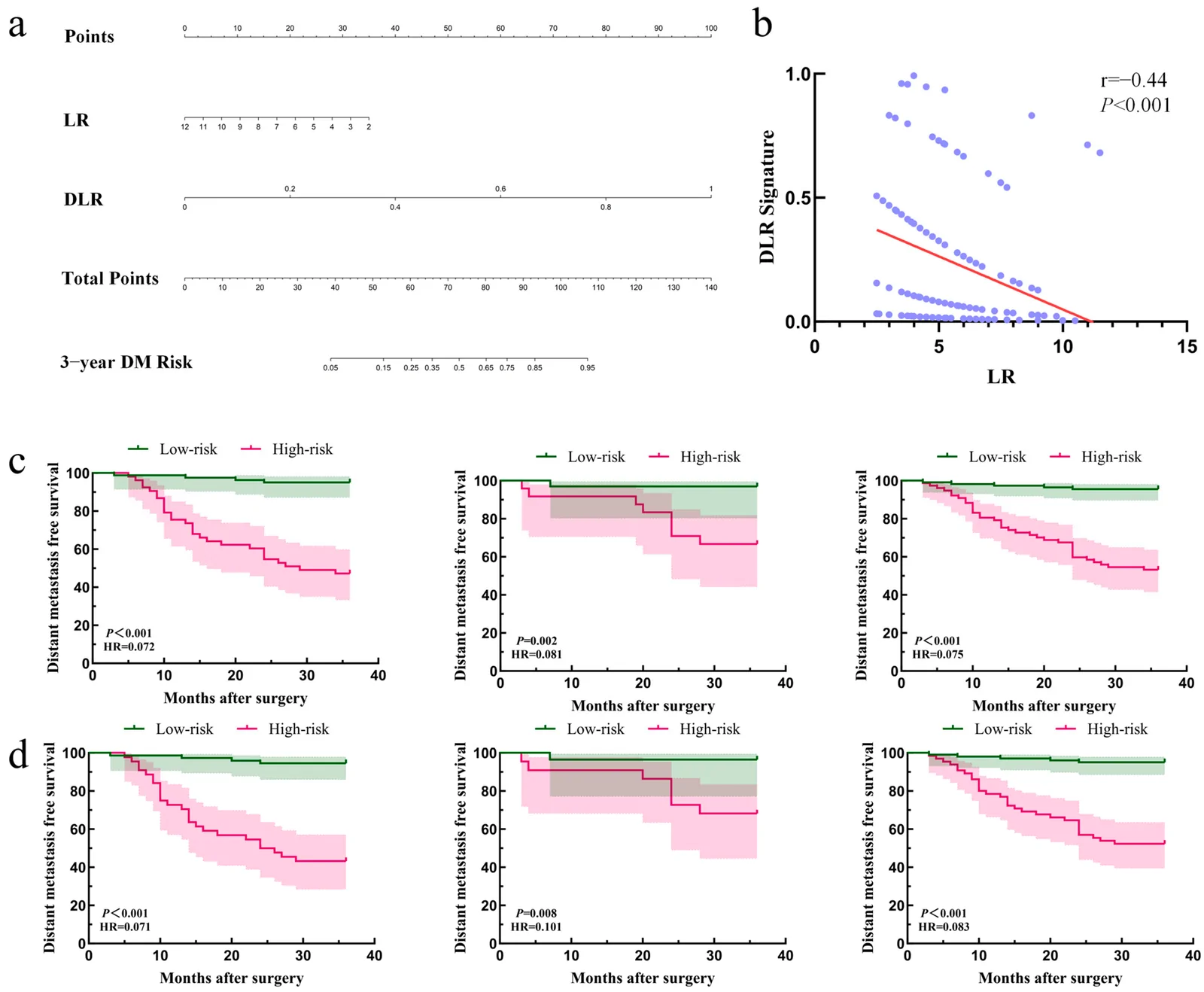
Nomogram construction, correlation analysis and Kaplan–Meier survival analysis for DMFS. (**a**) Nomogram for predicting 3-year distant metastasis risk. (**b**) Correlation analysis between DLR signature and LR. The red line denotes the linear regression fit. Pearson correlation coefficient r = −0.44, *p* < 0.001. (**c**) Kaplan–Meier DMFS curves in the training cohort. From left to right: high-versus low-risk groups; chemotherapy patients; non-chemotherapy patients; chemotherapy versus non-chemotherapy among low-risk patients; chemotherapy versus non-chemotherapy among high-risk patients. Number-at-risk tables are shown under each plot. (**d**) Kaplan–Meier DMFS curves in the validation cohort. From left to right: high- versus low-risk groups; chemotherapy patients; non-chemotherapy patients; chemotherapy versus non-chemotherapy among low-risk patients; chemotherapy versus non-chemotherapy among high-risk patients. Number-at-risk tables are shown under each plot.

Based on the LR and DLR signature, a nomogram was constructed to predict the 3-year distant metastasis risk for patients with RC (Figure 6a). A moderate negative correlation was detected between DLR signature and LR (r = −0.44, *p* < 0.001), indicating that higher LR values corresponded to lower DLR signature levels (Figure 6b). The nomogram risk score yielded good discriminative ability in both training and validation cohorts. The optimal cut-off value of 0.3 for the nomogram risk score was determined by the maximum Youden index from the training cohort. Patients were subsequently stratified into low-risk and high-risk subgroups according to this threshold.

Kaplan–Meier survival analyses were performed to evaluate DMFS differences across risk stratification and chemotherapy status in the training cohort (Figure 6c). A significant discrepancy in DMFS was observed between high-risk and low-risk patients. When further stratified by chemotherapy status, impaired DMFS was found in high-risk patients both among chemotherapy-treated and non-chemotherapy-treated individuals. We next explored the prognostic effect of chemotherapy within different risk subgroups. In Kaplan–Meier subgroup analyses, apparent DMFS differences were observed between patients with and without adjuvant chemotherapy among low-risk patients. Such survival patterns were not apparent in the high-risk subgroup.

Consistent findings were reproduced in the validation cohort (Figure 6d). Stratified Kaplan–Meier analyses confirmed worse DMFS in the high-risk group. Within chemotherapy-treated and non-chemotherapy-treated subgroups, high-risk patients consistently exhibited poorer DMFS outcomes. Again, apparent DMFS patterns associated with adjuvant chemotherapy were mainly observed among low-risk patients, while such patterns were not prominent in the high-risk subgroup.

## 4. Discussion

In the present retrospective study, we systematically constructed and internally validated five predictive models for 3-year DMFS risk stratification in RC, including clinicopathological, Rad, DTL, DLR, and integrated fusion models. Our core findings demonstrated that the fusion model combining pathological LR and DLR features achieved optimal discriminative power in both training (AUC = 0.911) and validation cohorts (AUC = 0.884). A significant negative linear correlation was observed between DLR signature and LR (r = −0.44, *p* < 0.001), revealing a potential immune-related biological basis for deep-learning radiomic biomarkers. Further stratified Kaplan–Meier survival analyses revealed observational survival patterns associated with adjuvant chemotherapy exposure within risk subgroups defined by the fusion model cut-off value of 0.3. Apparent DMFS differences related to adjuvant chemotherapy were observed among low-risk patients, whereas such survival patterns were not evident in high-risk patients. Collectively, this multimodal prediction system enables postoperative stratification of metastatic risk and may support hypothesis generation for future prospective studies as well as individualized postoperative surveillance for patients with RC.

Baseline clinicopathological comparisons of our enrolled cohorts provided preliminary evidence linking clinical and imaging indicators to DM. In the training cohort, longer tumors, lower LR scores, positive mrEMVI, advanced mrN stage, and a higher proportion of patients without chemotherapy were significantly associated with DM events (all *p* < 0.05). Among these candidate predictors, only LR and mrEMVI retained statistical significance in the validation cohort (both *p* < 0.05), highlighting their prognostic value across different patient populations. Multiple previous RC studies have established mrEMVI as a well-recognized risk factor for DM, as tumor cells invading extramural blood vessels facilitate hematogenous dissemination and form distant micro-metastases [12,13]. In contrast, LR quantifies the host antitumor immune response within the TME, consisting of four histological components: Crohn’s-like lymphoid aggregates, peritumoral lymphocytic clusters, intratumoral periglandular lymphocytic infiltration, and intratumoral tumor-infiltrating lymphocytes [14,15]. Multivariate Cox regression further confirmed LR as the independent protective prognostic factor for DMFS, independent of age, gender, tumor length, CEA, mrEMVI, mrT stage, mrN stage, and mrCRM. Patients with high LR exhibited significantly lower DM incidence, which aligns with prior immunopathology research demonstrating that abundant intratumoral immune infiltration enhances immune clearance of circulating tumor cells and suppresses metastatic progression [16,17,18]. Conventional clinicopathological indicators, such as tumor stage and serum CEA, failed to independently predict DM risk in our multivariate model, which may be attributed to their insufficient ability to reflect the heterogeneous antitumor immune status of individual tumors.

Given the clinical utility of predicting the 3-year DM risk, we designed and constructed five predictive models based on multidimensional features (the tumor LR, Rad features, DTL features, DLR features, and fusion features). The results showed that the fusion model integrating multidimensional features exhibited optimal comprehensive performance. This finding is highly consistent with the recent radiomics research trends in the field of tumor prognosis. For instance, some studies have confirmed that radiomic features based on MRI indirectly correlate with patients’ survival outcomes by reflecting the TME [19,20,21,22]. Jing et al. constructed and evaluated a combined model integrating radiomic features and clinical features, aiming to develop it as a non-invasive tool for the preoperative prediction of liver metastases after colorectal cancer surgery; this model exhibited favorable performance in both the training and validation cohorts, with corresponding AUCs of 0.761 [23]. Our multimodal fusion model targeting 3-year DMFS achieved higher AUC values (training 0.911; validation 0.884).

Currently, numerous studies have demonstrated that DLR models can predict the TME [24,25,26]. Correlation analysis revealed a moderate negative association between the DLR signature and LR (r = −0.44, *p* < 0.001), which may help partially explain the biological basis of the DLR model. Higher LR values reflected an immune-rich tumor microenvironment with abundant anti-tumor lymphocytes, which tended to correspond to lower DLR signature values derived from T_2_WI MRI sequences. This inverse association implies that the predictive performance of the DLR signature for DM risk may be partly driven by tumor LR status. The present study quantified the linear relationship between MRI deep learning features and histologically assessed antitumor immune response in RC, which could offer preliminary biological context to support the clinical utility of DLR signatures and reduce the “black-box” limitation of DLR biomarkers.

An important hypothesis-generating subgroup finding of this study is the set of observational differences associated with chemotherapy exposure across risk subgroups defined by the fusion model cut-off value of 0.3. Consistent patterns were seen in Kaplan–Meier survival analyses of both the training and validation cohorts: observational DMFS differences were apparent between patients who received and those who did not receive postoperative adjuvant chemotherapy among low-risk patients, while such survival patterns were not prominent within the high-risk subgroup. Mechanistically, low-risk tumors with high LR may correspond to an immune-active TME and relatively low intrinsic metastatic heterogeneity, which may render tumor micrometastases sensitive to fluoropyrimidine-based cytotoxic chemotherapy. In contrast, high-risk patients with low LR exhibit immune-suppressive TME and aggressive tumor phenotypes that may be associated with poorer outcomes following chemotherapy. Even after standard adjuvant chemotherapy, residual circulating tumor cells may progress to distant metastasis and result in unfavorable DMFS [27,28,29,30]. These exploratory subgroup observations allow the generation of hypotheses for future validation, and the multimodal risk stratification tool may support hypothesis generation for prospective studies. Of note, subgroup survival analyses demonstrated observational associations between adjuvant chemotherapy exposure and DMFS across nomogram-defined risk subgroups. Nevertheless, given the retrospective and non-randomized nature of chemotherapy allocation within this cohort, formal statistical testing for a treatment by risk group interaction was not conducted. These subgroup findings represent hypothesis-generating exploratory observations, rather than definitive proof of differential chemotherapy effects among different risk strata. Caution should be exercised when interpreting these associations, because treatment decisions were made at the discretion of treating clinicians, potentially leading to selection bias and unmeasured confounding. Our multimodal risk stratification tool may facilitate future prospective investigations. For the same reason, the inference that high-risk patients gain limited benefit from adjuvant chemotherapy requires careful interpretation owing to non-random treatment assignment.

This study has several limitations. First, the retrospective single-center design is prone to selection bias. The cohort split represents internal hold-out validation rather than true external validation. Multicenter studies using fully independent external cohorts are needed to validate our findings. Second, the internal validation cohort included only 57 patients with nine DM events. Predictive metrics such as AUC are sensitive to individual cases under low-event conditions. The broad 95% confidence interval for the fusion model validation cohort AUC (0.741–0.976) indicates substantial statistical uncertainty. Bootstrap optimism correction suggested mild over-optimism for training set performance. Moreover, only SVM hyperparameters were tuned via 5-fold cross-validation within the training cohort; full nested cross-validation across the whole feature selection pipeline was not performed due to computational constraints. Thus, model performance values should be considered preliminary and require confirmation in cohorts with larger DM event numbers. Third, chemotherapy assignment was non-randomized and clinician-driven, leaving chemotherapy-related confounders incompletely controlled. Observational subgroup survival associations were identified according to nomogram-based risk stratification. Formal treatment by risk group interaction testing was not pre-specified; therefore, these findings represent hypothesis-generating exploratory associations rather than definitive evidence of differential chemotherapy effects across risk strata. Fourth, there is methodological inconsistency: DTL features were extracted from the single largest axial tumor slice, whereas conventional radiomic features were derived from full 3D tumor volumes. Single-slice sampling may miss intratumoral heterogeneity. Future work should evaluate multi-slice or volumetric 3D deep learning radiomics.

## 5. Conclusions

In this study, we constructed and validated a fusion nomogram integrating pathological LR and MRI DLR signatures to predict 3-year DMFS risk in patients with RC. The model maintained consistent predictive performance across internal training and validation cohorts. The moderate negative correlation observed between DLR signatures and LR provides preliminary immune-related evidence to contextualize DLR biomarkers. Observational subgroup survival associations were identified according to nomogram-based risk stratification. This postoperative multimodal tool integrating postoperative histopathological and preoperative MRI-derived signatures may facilitate personalized postoperative surveillance and may act as a supportive reference for risk stratification and individualized clinical management of RC. Nevertheless, these findings should be considered preliminary, and further validation in large-scale independent multicenter cohorts is required before broad clinical implementation.

## Figures and Tables

**Table 1:** Clinicopathologic characteristics of patients.

Feature	Train Total (*n* = 133)	Train Cohort			Validation Total (*n* = 57)	Validation Cohort			*p* Value
		Non-DM (*n* = 101)	DM (*n* = 32)	*p* Value		Non-DM (*n* = 48)	DM (*n* = 9)	*p* Value	
Age	62.11 ± 8.60	61.49 ± 7.70	64.09 ± 10.87	0.225	60.61 ± 11.08	61.10 ± 11.00	58.00 ± 11.78	0.553	0.822
Gender				0.809				0.7	0.877
F	46 (34.59)	36 (35.64)	10 (31.25)		19 (33.33)	15 (31.25)	4 (44.44)		
M	87 (65.41)	65 (64.36)	22 (68.75)		38 (66.67)	33 (68.75)	5 (55.56)		
Length	4.38 ± 1.33	4.24 ± 1.29	4.83 ± 1.38	0.013	4.25 ± 1.04	4.17 ± 1.04	4.72 ± 0.94	0.118	0.675
LR	5.31 ± 1.78	5.51 ± 1.74	4.69 ± 1.82	0.004	5.31 ± 2.00	5.59 ± 2.06	3.81 ± 0.39	0.006	0.687
CEA	10.82 ± 18.2	10.26 ± 16.50	12.57 ± 22.97	0.998	9.39 ± 19.20	10.30 ± 20.78	4.50 ± 3.35	0.922	0.741
Chemotherapy				0.01				0.122	0.942
no	37 (27.82)	21 (20.79)	16 (50.00)		16 (28.07)	11 (22.92)	5 (55.56)		
yes	96 (72.18)	80 (79.21)	16 (50.00)		41 (71.93)	37 (77.08)	4 (44.44)		
mrEMVI				0.001				0.011	0.846
Negative	88 (66.17)	75 (74.26)	13 (40.62)		37 (64.91)	35 (72.92)	2 (22.22)		
Positive	45 (33.83)	26 (25.74)	19 (59.38)		20 (35.09)	13 (27.08)	7 (77.78)		
mrTstage				0.159				0.101	0.269
2	26 (19.55)	23 (22.77)	3 (9.38)		16 (28.07)	16 (33.33)	0		
3–4	107 (80.45)	78 (77.23)	29 (90.62)		41 (71.93)	32 (66.67)	9 (100.00)		
mrNstage				0.049				0.1	0.82
0	80 (60.15)	66 (65.35)	14 (43.75)		36 (63.16)	33 (68.75)	3 (33.33)		
1–2	53 (39.85)	35 (34.65)	18 (56.25)		21 (36.84)	15 (31.25)	6 (66.67)		
mrCRM				0.534				1.0	0.758
Negative	103 (77.44)	80 (79.21)	23 (71.88)		46 (80.70)	39 (81.25)	7 (77.78)		
Positive	30 (22.56)	21 (20.79)	9 (28.12)		11 (19.30)	9 (18.75)	2 (22.22)		

LR: lymphocytic reaction; CEA: carcinoembryonic antigen; EMVI: extramural vascular invasion; CRM: circumferential resection margin.

## Data Availability

The datasets generated and analyzed during the current study are available from the corresponding author upon reasonable request.

## Supplementary Materials

The following supporting information can be downloaded at: https://www.mdpi.com/article/10.3390/cancers18183020/s1, File S1. Full list of 1198 conventional radiomic features extracted by PyRadiomics; File S2. Details of 2048 DTL features extracted using the prebuilt ResNet50 convolutional neural network; Table S1. Scanning parameters; Table S2; Interobserver agreement for MRI-derived features and pathological lymphocytic reaction scoring; Table S3. Final 12 Predictive Features Selected by LASSO Regression for the DLR Model; Table S4. Performance metrics of all prediction models in the training and test cohorts.

## Abbreviations

AUC	Area under curve
CEA	Carcinoembryonic antigen
CRM	Circumferential resection margin
CI	Confidence interval
DTL	Deep transfer learning
DLR	Deep learning radiomics
DM	Distant metastasis
DMFS	Distant-metastasis-free survival
DCA	Decision curve analysis
EMVI	Extramural vascular invasion
HR	Hazard ratio
ICCs	Intraclass correlation coefficients
LASSO	Least absolute shrinkage and selection operator
Rad	Radiomics
RC	Rectal cancer
ROC	Receiver operating characteristic
ROI	Region of interest
TME	Tumor microenvironment
