# Supplementary material for: Lymphocytic Reaction Combined with MRI Deep Learning Radiomics in Risk Stratification of Distant Metastases in Rectal Cancer
Source: Cancers (Basel). 2026 Sep 17;18(18):3020. doi: 10.3390/cancers18183020 (PMC13605595; doi:10.3390/cancers18183020)
Supplement: Supplementary file 1 [file cancers-18-03020-s001.zip › Table S1.pdf]

Table S1 Scanning parameters

Scanner	TR (ms)	TE (ms)	FOV (mm)	Matrix	Bandwidth (kHz)	NEX	Thickness (mm)	Gap (mm)
Achieva 1.5T	5394	100	240	240×187	246	1	5	0.5
Skyra 3.0T	4700	98	250	256×320	200	1	4.5	0.5
