# Supplementary material for: Lymphocytic Reaction Combined with MRI Deep Learning Radiomics in Risk Stratification of Distant Metastases in Rectal Cancer
Source: Cancers (Basel). 2026 Sep 17;18(18):3020. doi: 10.3390/cancers18183020 (PMC13605595; doi:10.3390/cancers18183020)
Supplement: Supplementary file 1 [file cancers-18-03020-s001.zip › Table S2.pdf]

Table S2 Interobserver agreement for MRI-derived features and pathological lymphocytic reaction scoring

Category	Evaluation item	Statistical index	Result
MRI Tumor Segmentation	Radiomic features	ICC	Range: 0.79 – 0.91 (median = 0.85; mean ± SD = 0.86 ± 0.06)
	DTL features	ICC	Range:0.78–0.90 (median = 0.82; mean ± SD = 0.82 ± 0.04)
Pathological LR Scoring	Crohn's-like lymphocytic reaction	Weighted Kappa coefficient (κ)	0.81(95% CI: 0.73–0.87)
	Peritumoral lymphocytic reaction	Weighted Kappa coefficient (κ)	0.79(95% CI: 0.69–0.86)
	Intratumoral periglandular reaction	Weighted Kappa coefficient (κ)	0.84(95% CI: 0.76–0.90)
	Tumor-infiltrating lymphocytes	Weighted Kappa coefficient (κ)	0.86(95% CI: 0.78–0.91)
Discrepancy and Consensus (total n=190)	MRI discrepancy	Cases (%)	13 (6.8%)
	Pathological LR scoring discrepancy	Cases (%)	9 (4.7%)
	Consensus resolution for discrepant cases	-	100% resolved by joint review
