# Supplementary material for: Lymphocytic Reaction Combined with MRI Deep Learning Radiomics in Risk Stratification of Distant Metastases in Rectal Cancer
Source: Cancers (Basel). 2026 Sep 17;18(18):3020. doi: 10.3390/cancers18183020 (PMC13605595; doi:10.3390/cancers18183020)
Supplement: Supplementary file 1 [file cancers-18-03020-s001.zip › Table S3.pdf]

Table S3 Final 12 Predictive Features Selected by LASSO Regression for the DLR Model

Feature Name	Feature Category
wavelet_LHH_glszm_SizeZoneNonUniformity	Wavelet-domain radiomic feature
wavelet_LLH_glszm_SmallAreaEmphasis	Wavelet-domain radiomic feature
DL_7	Deep learning (DTL) feature
log_sigma_2_0_mm_3D_glszm_ZoneEntropy	Filter-domain radiomic feature
log_sigma_4_0_mm_3D_glcm_Idn	Filter-domain radiomic feature
wavelet_LHL_glrlm_LongRunHighGrayLevelEmphasis	Wavelet-domain radiomic feature
DL_164	Deep learning (DTL) feature
wavelet_HLL_glszm_SmallAreaLowGrayLevelEmphasis	Wavelet-domain radiomic feature
DL_51	Deep learning (DTL) feature
wavelet_LLH_glcm_Imc2	Wavelet-domain radiomic feature
wavelet_HHL_glcm_Correlation	Wavelet-domain radiomic feature
wavelet_HHH_firstorder_Minimum	Wavelet-domain radiomic feature
