# Supplementary material for: Lymphocytic Reaction Combined with MRI Deep Learning Radiomics in Risk Stratification of Distant Metastases in Rectal Cancer
Source: Cancers (Basel). 2026 Sep 17;18(18):3020. doi: 10.3390/cancers18183020 (PMC13605595; doi:10.3390/cancers18183020)
Supplement: Supplementary file 1 [file cancers-18-03020-s001.zip › Table S4.pdf]

Supplementary Table S4. Performance metrics of all prediction models in the training and test cohorts

Model	Cohort	AUC	95% CI	Sensitivity	Specificity	PPV	NPV	PR-AUC
Clinic	Training	0.788	0.698-0.877	0.650	0.720	0.607	0.755	0.631
	Test	0.777	0.640-0.914	0.630	0.680	0.563	0.738	0.572
Rad	Training	0.855	0.789-0.921	0.760	0.753	0.673	0.825	0.740
	Test	0.778	0.573-0.982	0.740	0.710	0.632	0.803	0.610
DTL	Training	0.755	0.639-0.870	0.660	0.700	0.595	0.755	0.622
	Test	0.755	0.533-0.977	0.625	0.650	0.543	0.722	0.560
DLR	Training	0.897	0.844-0.951	0.820	0.793	0.726	0.869	0.801
	Test	0.838	0.707-0.969	0.775	0.733	0.660	0.830	0.690
Combined	Training	0.911	0.853-0.968	0.880	0.847	0.793	0.914	0.858
	Test	0.884	0.777-0.992	0.850	0.800	0.739	0.889	0.768

AUC, area under the receiver-operating characteristic curve; CI, confidence interval; PPV, positive predictive value; NPV, negative predictive value; PR-AUC, area under the precision-recall curve.
